# Development of a Recombinant Pichinde Virus-Vectored Vaccine against Turkey Arthritis Reovirus and Its Immunological Response Characterization in Vaccinated Animals

**DOI:** 10.3390/pathogens10020197

**Published:** 2021-02-13

**Authors:** Pawan Kumar, Tamer A. Sharafeldin, Rahul Kumar, Qinfeng Huang, Yuying Liang, Sagar M. Goyal, Robert E. Porter, Hinh Ly, Sunil K. Mor

**Affiliations:** 1 Department of Veterinary Population Medicine, College of Veterinary Medicine, University of Minnesota, Saint Paul, MN 55108, USA; pkbagri.vets@gmail.com (P.K.); shara022@umn.edu (T.A.S.); kumar509@umn.edu (R.K.); goyal001@umn.edu (S.M.G.); porte349@umn.edu (R.E.P.); 2 Department of Animal Biotechnology, College of Veterinary Sciences, Lala Lajpat Rai University of Veterinary & Animal Sciences, Hisar, Haryana 125001, India; 3 Department of Pathology, Faculty of Veterinary Medicine, Zagazig University, Zagazig, Sharkia 44511, Egypt; 4 Department of Veterinary Pathology, College of Veterinary Science and Animal Husbandry, Pandit Deen Dayal Upadhyaya Veterinary Science University and Cattle Research Institute, Mathura 281001, India; 5 Department of Veterinary and Biomedical Sciences, College of Veterinary Medicine, University of Minnesota, Saint Paul, MN 55108, USA; huangq@umn.edu (Q.H.); liangy@umn.edu (Y.L.)

**Keywords:** Pichinde virus, recombinant vaccine, subunit vaccine, viral vectored vaccine, turkey arthritis reovirus, sigma C, sigma B

## Abstract

Vaccination may be an effective way to reduce turkey arthritis reovirus (TARV)-induced lameness in turkey flocks. However, there are currently no commercial vaccines available against TARV infection. Here, we describe the use of reverse genetics technology to generate a recombinant Pichinde virus (PICV) that expresses the Sigma C and/or Sigma B proteins of TARV as antigens. Nine recombinant PICV-based TARV vaccines were developed carrying the wild-type S1 (Sigma C) and/or S3 (Sigma B) genes from three different TARV strains. In addition, three recombinant PICV-based TARV vaccines were produced carrying codon-optimized S1 and/or S3 genes of a TARV strain. The S1 and S3 genes and antigens were found to be expressed in virus-infected cells via reverse transcriptase polymerase chain reaction (RT-PCR) and the direct fluorescent antibody (DFA) technique, respectively. Turkey poults inoculated with the recombinant PICV-based TARV vaccine expressing the bivalent TARV S1 and S3 antigens developed high anti-TARV antibody titers, indicating the immunogenicity (and safety) of this vaccine. Future in vivo challenge studies using a turkey reovirus infection model will determine the optimum dose and protective efficacy of this recombinant virus-vectored candidate vaccine.

## 1. Introduction

Turkey arthritis reoviruses (TARVs) re-emerged in 2011 in Minnesota and other states in the United States (US) [1,2,3]. The viral genome consists of 10 segments of dsRNA grouped into large (L1, L2, L3), medium (M1, M2, M3), and small (S1, S2, S3, S4) segments according to their migration pattern on polyacrylamide gel electrophoresis [4,5]. The genome has 12 open reading frames (ORFs), which encode for eight structural and four nonstructural proteins. The proteins encoded by L, M, and S genes are lambda (λ), mu (m), and sigma (σ), respectively [5]. The S1 and S3 segments translate into σC (cell attachment) and σB (outer capsid) proteins, respectively. The σC protein is a minor outer capsid protein of 326 amino acids, which has been identified as cell attachment protein responsible for virus entry into the host cell [6]. The σC protein mediates virus entry into the host cell via the Caveolin-1-mediated and dynamin-2-dependent endocytic pathway [7]. The σC protein is also known to induce apoptosis [8]. The C-terminal fragment of σC (residues 151–326) contains the receptor-binding globular domain [9]. The σC protein is the main immunogenic surface protein that possesses both type- and broad-specific epitopes, and it can elicit reovirus-specific neutralizing antibodies in the infected host [10]. The σB protein of 367 amino acids is a major component of the viral outer capsid that contains a group-specific neutralizing epitope [11]. Subunit vaccines for chicken arthritis reovirus (CARV) are available that contain either a partial fragment of σC protein [12] or a full-length σC protein alone [13] or in combination with σB protein [14]. The recombinant σC protein as a subunit vaccine has been used against duck reovirus [15]. Hence, σC and σB proteins have been commonly used in the development of subunit vaccines against avian reovirus infection in different avian species. Since TARVs are similar to CARVs in terms of their genomic structure, segment sizes, and open reading frames, the σC and σB of TARV should be considered in the development of mono- or multivalent virus vectored vaccines against TARV.

TARV-infected turkeys display clinical lameness due to tenosynovitis and arthritis, resulting in huge economic losses mainly due to culling. No commercial vaccine is available to protect turkey flocks from the emerging TARV strains. Some turkey producers rely on the use of autogenous vaccines. However, the evolving nature of the virus creating new divergent strains poses a challenge for regular update of the vaccines. Additionally, there is no live vaccine available that can be used for priming before boosting breeder turkeys with injectable killed vaccines. 

Using a live and safe vectored vaccine to deliver TARV antigens is a reasonable alternative to live reovirus vaccines. Recently, a Pichinde virus (PICV) vector was developed that safely and effectively delivered the model antigens, the influenza viral hemagglutinin (HA), and nucleoprotein (NP) [16]. This arenavirus was first isolated from its natural host *Oryzomys albigularis* (rice rats) in the Pichinde valley of Colombia, South America [17]. Arenaviruses are enveloped RNA viruses with a bisegmented genome and are known to target dendritic cells and macrophages at the early stages of infection, making them a potentially powerful vaccine vector [18,19,20,21]. 

Using the reverse genetics technique, a live recombinant PICV (strain 18) with a trisegmented RNA genome (rP18tri) was developed to carry and express up to two foreign genes. One of the foreign genes could be the green fluorescent protein (GFP) that can be used to mark virus-infected cells in cell culture. rP18tri has been shown to be attenuated both in vitro and in vivo and has the ability to induce cell-mediated and humoral immune responses. For example, mice immunized with recombinant PICV–hemagglutinin (PICV–HA), expressing the modeled influenza hemagglutinin protein, developed a strong humoral response against HA that afforded complete protection against a lethal avian influenza virus infection [16]. 

The present study was undertaken to develop recombinant PICV vaccines expressing TARV antigenic Sigma C (σC) and/or Sigma B (σB) proteins. In addition, the vaccines were administered to turkey poults to determine their safety and efficacy. This is a “proof-of-concept” study for the development and recovery of recombinant PICV–TARV viruses, as well as for testing the safety and immunogenic properties of the turkey reovirus σC and σB protein(s) in vivo.

## 2. Materials and Methods

### 2.1. Cells and Viruses

QT-35 cells were grown in Dulbecco’s modified Eagle’s medium (DMEM) (Sigma-Aldrich, St. Louis, MO, USA) containing 10% fetal bovine serum (FBS) and 50 ug/mL penicillin–streptomycin. Baby hamster kidney (BHK-21) cells, BSRT7-5 cells (BHK-21 cells stably expressing T7 RNA polymerase), and male leghorn chicken (LMH) cells were grown in Eagle’s minimal essential medium (MEM) (Sigma-Aldrich) that contained 10% FBS, 1 ug/mL gentamicin, and 50 ug/mL penicillin–streptomycin. Media for BSRT7-5 cells was also supplemented with 1 µg/mL geneticin (Invitrogen-Life Technologies, Carlsbad, CA, USA). Three strains of turkey arthritis reovirus (SKM73, SKM95, and SKM121) isolated in QT-35 cells from tendons of lame turkeys were used. These viruses were selected on the basis of their pathogenicity and genomic characterization.

### 2.2. Pichinde Virus Plasmids

Three plasmids were used: (i) pP18S1-GPC/MCS, which encodes the glycoprotein GPC and a multiple cloning site (MCS) to clone the gene of interest; (ii) pP18S2-MCS/NP, which encodes the nucleoprotein NP and an MCS; (iii) pP18L plasmid (the L plasmid), which expresses the full-length antigenomic strand of the rP18L segment under the control of the T7 promoter and does not contain any specific site to clone foreign genes [16]. 

### 2.3. Preparation of Vectors and Gene Inserts

Genomic RNA was isolated from three TARV isolates (SKM73, SKM95, and SKM121). The full-length open reading frames (ORFs) of S1 and S3 genomic segments of these viruses were amplified. Additionally, the S1 and S3 ORF sequences of SKM121 were codon-optimized for expression in mammalian cells, commercially custom-synthesized, and cloned into a pUC vector (Genewiz, South Plainfield, NJ, USA). The primary sequences of these genes are provided in Supplementary Materials (Figure S1). The complementary DNA (cDNA) was synthesized using random primers and SuperScript™ IV First-Strand Synthesis (Thermo Fisher Scientific, Catalog#18091200, Waltham, MA, USA) and was PCR-amplified using specific cloning primers (Table 1) and Phusion® High-Fidelity PCR Master Mix with HF Buffer (NEB Catalog#M0531S, Ipswich, MA, USA). The *Nhe*I and Kozak sequences were added in the forward primer so that these features were included in the amplified product. Similarly, *Xho*I and sequence tags (FLAG tag in S1 and HA tag in S3 gene) were added to the reverse primer. The reaction conditions were as follows: 98 °C for 30 s (initial denaturation); five cycles of denaturation at 98 °C for 10 s, annealing at 66 °C for 30 s, and extension at 72 °C for 1 min; 30 cycles of denaturation at 98 °C for 10 s, annealing at 72 °C for 30 s, and extension at 72 °C for 1 min; final extension at 72 °C for 7 min and held at 4 °C. The PCR-amplified products (S1 and S3 genes of all three isolates) and the plasmids of the PICV (pP18S1-GPC/MCS and pP18S2-MCS /NP) were restriction enzyme-digested (*Nhe*I and *Xho*I, NEB) and gel-purified using the QIAquick gel extraction kit (Qiagen Catalog#28704, Germantown, MD, USA). The codon-optimized versions of ORFs were extracted from the pUC vector via restriction double digestion.

### 2.4. Cloning and Transfection

The S1 and S3 gene inserts were ligated in the MCS region of plasmids pP18S2-MCS/NP and pP18S1-GPC/MCS, respectively, using 5 U/µL of T4 DNA ligase (ThermoFisher Scientific, Catalog#EL0011). The ligation reaction mix was used to transform competent bacterial cells (DH5α) followed by selection using ampicillin antibiotic. All plasmids (plasmid pP18L, pP18S1-GPC/GFP, pP18S2-GFP/NP, and recombinant plasmids pP18S1-GPC/S3 and pP18S2-S1/NP) were isolated using the plasmid midi prep kit (Sigma-Aldrich). Recombinant plasmids were PCR-confirmed for reovirus genes and sequence-confirmed for correct orientation and reading frame. The recombinant plasmids were used to transfect BSRT7-5 cells in various combinations (Table 2) using Lipofectamine™ 3000 transfection reagent (ThermoFisher, Catalog#L3000008) following the manufacturer’s instruction with minor modifications. Briefly, BSRT7-5 cells were grown in six-well plates to 80% confluency. Four hours before transfection, the cells were washed, and fresh antibiotic-free medium was added. For transfection, 8 µL of P3000 reagent, 2 µg of L plasmid and 1 µg each of S1 and S2 plasmids were diluted in 250 µL of Opti-MEM (Invitrogen-Life Technologies) and incubated for 15 min at room temperature. In another tube, 10 µL of lipofectamine was diluted in 250 µL of Opti-MEM. Both mixtures were combined, followed by incubation at room temperature for 20 min to prepare DNA–lipid complexes. The cells were transfected with the resultant mixture, and MEM was changed after 4 h to remove the toxic lipofectamine. Then, at 48, 72, and 96 h post transfection, cell supernatants were collected and stored at −80 °C. Different monovalent and bivalent vector viruses were generated as detailed in Table 2. The resultant viral recovery was confirmed by observing the green fluorescence of GFP in inoculated cell culture. The rescued virus was then grown in BHK-21 cells, and the GFP green fluorescence was observed. The expression of reovirus genes by the recombinant PICV vaccine virus was verified by RT-PCR assay.

### 2.5. Detection of Reovirus Antigenic Proteins

The expression of σC and σB proteins by recombinant trisegmented PICV vaccine viruses in BHK-21 cells was verified using a direct fluorescence antibody (DFA) assay. The BHK-21 cells were inoculated with the recombinant PICVs and at 96 h post infection (hpi); then, the cells were harvested, plated on 12-chamber slides, and dried for 2 h. Cells were then fixed in acetone for 2 h followed by the addition of polyclonal Fluorescein isothiocyanate (FITC)-conjugated anti-avian reovirus antibodies (National Veterinary Services Laboratory, Ames, IA, USA, Reagent#680-ADV). After incubation at 37 °C in a CO_2_ incubator for 2 h, counterstaining was done using 0.1% Evan’s blue biological stain (EBBS). The slides were then mounted and examined under a fluorescent microscope to observe FITC green fluorescence, which was indicative of avian reovirus protein expression by the vaccine viruses. 

### 2.6. Vaccination Experiment

Four groups of turkey poults (five birds/group) were inoculated with 0.3 mL of the following recombinant PICVs containing codon-optimized gene segments of TARV-SKM121: PICV-based monovalent TARV-S1, monovalent TARV-S3, bivalent TARV-S1/S3, and PICV-control without any TARV segment insertion, having 3 × 10^5^ plaque forming unit/mL (PFU/mL) via oral route at 1 week of age. Birds in all groups were given a booster dose (0.3 mL, 3 × 10^5^ PFU/mL) with respective vaccine viruses at 3 weeks of age via intranasal (I/N) route. Blood samples were collected at 3 and 5 weeks of age. The birds were examined daily for any overt clinical signs or mortality. Birds displaying signs of severe illness were euthanized according to the guidelines laid by the Institutional Animal Care and Use Committee (IACUC) and research animal resources (RAR), University of Minnesota. At the end of the experiment, all birds were euthanized, and necropsy was done to detect the development of any gross lesions. 

### 2.7. Serum Neutralization Assay

Sera were separated from the collected blood samples and subjected to serum neutralization assay against virus strain TARV-SKM121. Briefly, the heat-inactivated serum samples were fourfold serially diluted in a 96-well plate, and 25 µL of reovirus preparation (100 Median Tissue Culture Infectious Dose (TCID50)) was added to all wells except negative control wells. Subsequently, the virus–serum mixture was incubated at 37 °C for 1 h before adding it onto a freshly seeded primary hepatocellular carcinoma epithelial cell from a male leghorn chicken (LMH) (5 × 10^5^ cells/well) with 10% fetal calf serum and incubated for 4–5 days. Virus-infected and uninfected cells were used as positive and negative controls, respectively. Virus controls, cell controls, and serum controls were included in each plate. The plates were observed daily for the appearance of cytopathic effects (CPEs). When a cytopathic effect was observed, the medium was removed, and the cells were stained with a 1% crystal violet solution prepared in 10% buffered formalin for 2–3 min, followed by two to three gentle washes with warm tap water. Plates were allowed to dry, and the titer was recorded as the reciprocal of the highest dilution of serum that inhibited at least 50% cell destruction. Serum neutralization titers among different groups were subjected to statistical analysis using nonparametric Kruskal–Wallis test followed by Mann–Whitney U test for testing statistical significance at *p* < 0.05. 

## 3. Results

### 3.1. Cloning of Reovirus Genes into PICV Plasmids

**Figure 1:**
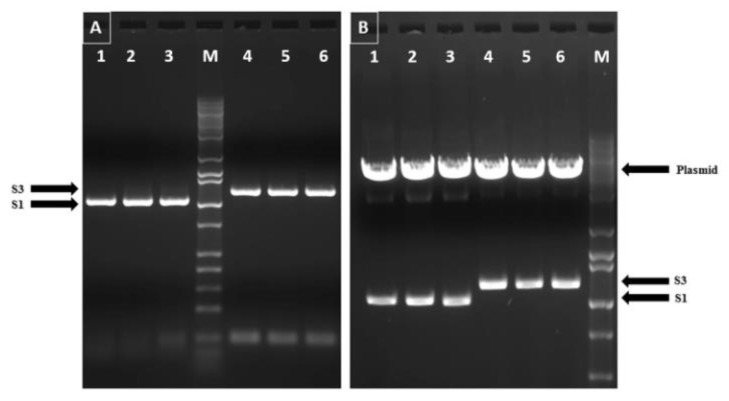
Confirmation of turkey arthritis reovirus genes (S1 and S3) in the recombinant PICV vector by restriction enzyme (RE) double digestion: (**A**) RT-PCR amplification of full-length S1 (σC) and S3 (σB) open reading frames of turkey arthritis reovirus. Lanes 1, 2, and 3: S1 gene of TARV strains SKM73, SKM95, and SKM121, respectively (1,031 bp); M: Marker; Lanes 4, 5, and 6: S3 gene of TARV strains SKM73, SKM95, and SKM121, respectively (1,157 bp); (**B**) Restriction enzyme (RE) double digestion confirms the presence of S1 and S3 genes of TARV in recombinant PICV plasmids. Lanes 1, 2, and 3: RE double digestion of recombinant pP18S2-S1/NP plasmid yielding plasmid backbone and codon-optimized insert of 1031 bp of S1 (σC); Lanes 4, 5, and 6: RE double digestion of recombinant pP18S1-GPC/S3 plasmid yielding plasmid backbone and codon-optimized insert of 1157 bp of S3 (σB); M: Marker.

The RT-PCR amplification of S1 (Sigma C) and S3 (Sigma B) ORFs yielded the expected product sizes of 1031 bp and 1157 bp, respectively (Figure 1A). These products were gel-purified and cloned into pP18S2-MCS /NP and pP18S1-GPC/MCS, respectively. Restriction enzyme double digestion confirmed the presence of reovirus genes in recombinant PICV plasmids (Figure 1B). Sanger sequencing confirmed the absence of unintended mutations in the cloned viral gene, as well as their correct reading frame and correct orientation in the vector backbones (data not shown).

### 3.2. Plasmid Transfection and Virus Rescue

**Figure 2:**
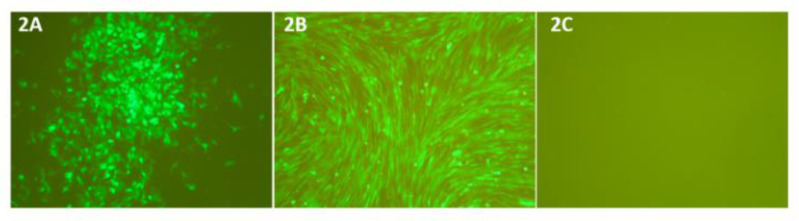
Transfection and infection of cells with plasmids and recombinant PICV-based TARV vaccine viruses, respectively. (**A**) GFP expression in BSRT7-5 cells was observed under a fluorescence microscope following transfection of cells with pP18S1-GPC/GFP and pP18S2- S1/NP having a codon-optimized S1 gene insert, as shown in Table 2, to generate a recombinant PICV-based TARV vaccine number 10. (**B**) GFP expression in BHK-21 cells infected with supernatant of recombinant monovalent PICV-based TARV vaccine number 10 at 96 hours post infection. (**C**) BSRT7-5 cells transfected with pP18S1-GPC/S3 and pP18S2- S1/NP having codon-optimized S1 and S3 gene inserts to generate a recombinant bivalent PICV-based TARV vaccine (recombinant vector virus number 12 of Table 2) that lacks the green fluorescence.

Viable recombinant PICVs were rescued successfully following transfection of BSRT7-5 cells with the three plasmids in various combinations, as shown in Table 2. The GFP expression was observed at 48–72 h post transfection in cells transfected with at least one GFP-containing plasmid (Figure 2A) (all monovalent vaccines in Table 2). The GFP-expressing foci increased in size over the time course of transfection. The supernatants were collected from transfected BSRT7-5 cells and were used to infect BHK21 cells. Strong GFP expression was detected in infected BHK21 cells at 24–48 hpi using fluorescence microscopy (Figure 2B), indicating the rescue of viable PICV-based TARV vaccines (viruses). At every rescue attempt, we obtained infectious viruses at 48–72 h post transfection. The recombinant viruses showed minor GFP fluorescence in QT-35 and LMH cells at 96 hpi. As expected, the bivalent PICV-based TARV vaccines carrying two TARV genes on both PICV plasmids did not produce any green fluorescence (Figure 2C).

### 3.3. Recombinant PICVs Expressing Reovirus Antigens

**Figure 3:**
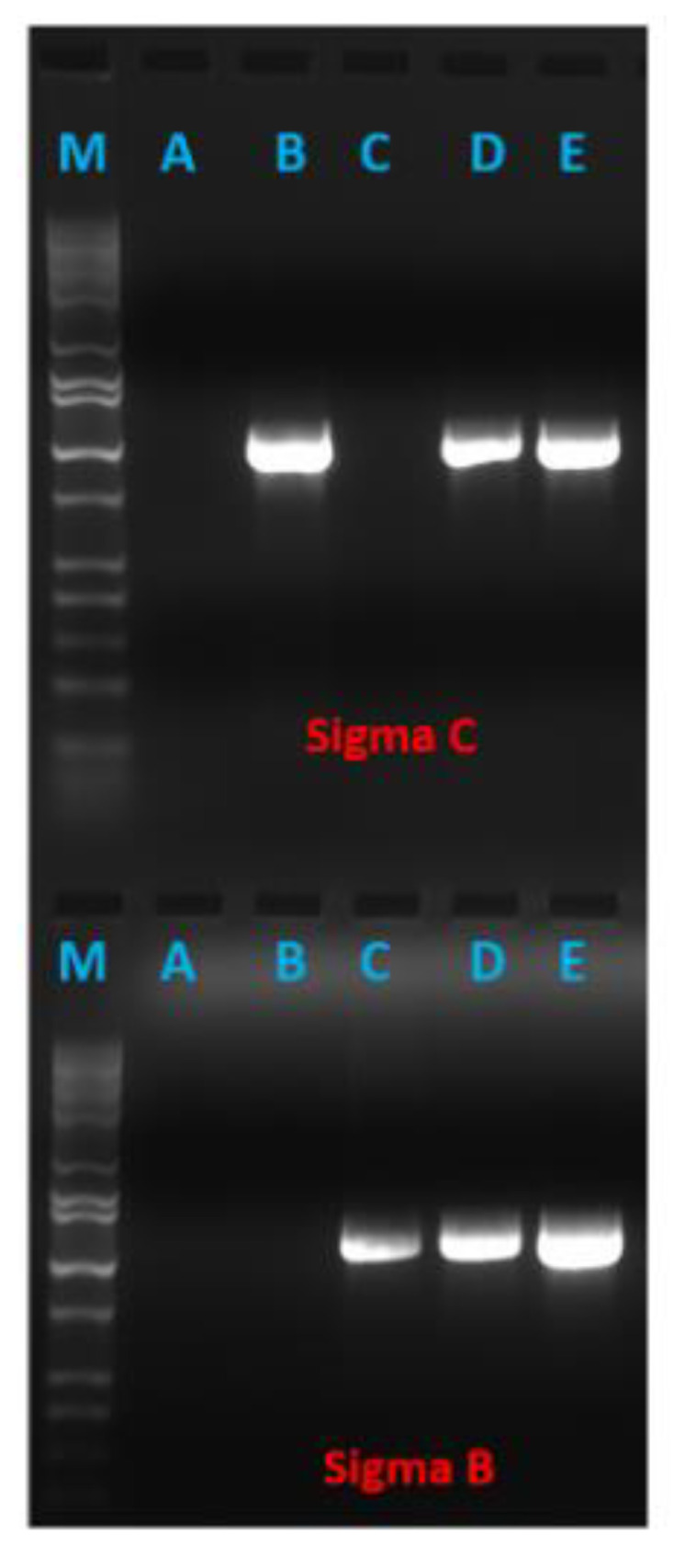
RT-PCR assay indicating the expression of turkey reovirus antigens at the messenger RNA (mRNA) level by recombinant PICV-based TARV vaccines. Lanes: M, marker; A, recombinant PICV carrying only GFP gene; B, recombinant PICV carrying σC gene; C, recombinant PICV carrying σB gene; D, recombinant PICV carrying σC and σB genes (after 24 h of infection); E, recombinant PICV carrying σC and σB genes (after 48 h of infection).

**Figure 4:**
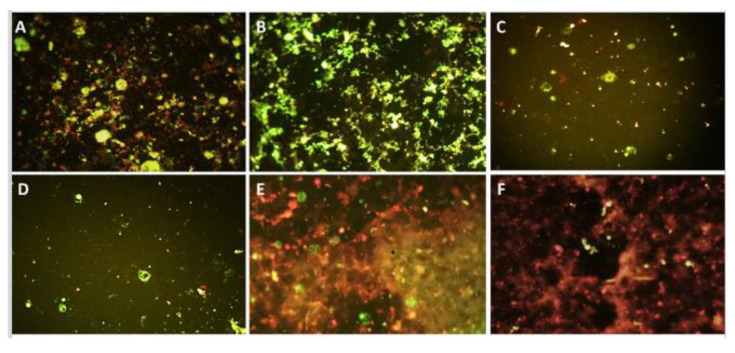
The recombinant PICV-based TARV vaccines expressing turkey arthritis reovirus antigens. Fluorescence in BHK-21 cells (green fluorescent cells) based on direct fluorescent antibody test indicates TARV protein expression. (**A**) SKM121 wild-type virus control; (**B**) PICV-based TARV vaccine carrying bivalent SKM121 codon-optimized S1 and S3 sequences; (**C**) PICV-based TARV vaccine carrying bivalent SKM121 wild-type S1 and S3 sequences; (**D**) PICV-based TARV vaccine carrying monovalent SKM121 wild-type sequence; (**E**) PICV-based TARV vaccine carrying monovalent SKM121 codon-optimized sequence; (**F**) negative control.

Strong GFP expression by infected BHK21 cells indicated the successful rescue of recombinant PICV-based TRAV vaccines (viruses). The supernatant from infected BHK21 cells (passages P1, P2, and up to P3) was used to detect reovirus genes by RT-PCR. The results confirmed the presence of both viral genes in bivalent PICV-based TARV vaccines and either S1 or S3 gene in the monovalent vaccine viruses. The RT-PCR amplification indicated TARV antigen expression at the messenger RNA (mRNA) level, where the supernatant harvested at 48 h post infection showed significantly higher amplification than the supernatant harvested at 24 h post infection (Figure 3). To verify the expression of reovirus antigenic proteins (σC and σB) by the recombinant PICVs, we infected BHK21 cells with transfection supernatant and, then, at 96 hpi, conducted a direct fluorescence assay (DFA) using polyclonal FITC-conjugated anti-avian reovirus antibodies. The PICVs grown on BHK-21 showed varying degrees of fluorescence (Figure 4). The monovalent and bivalent PICV-based TARV vaccines that contained S1 and/or S3 showed fluorescence in BHK-21 cells (Figure 4A–E). Although we did not quantify the amount of fluorescence, PICVs containing SKM121 gene segments showed a remarkably higher degree of fluorescence, particularly the bivalent PICV-based TARV vaccine that contained codon-optimized S1 and S3 segments (Figure 4B). Minimal fluorescence was observed in negative controls (cells that contained rescued PICV without any TARV segment) (Figure 4F).

### 3.4. Clinical Signs and Necropsy

Birds inoculated with the PICV-based TARV vaccines (monovalent and bivalent) did not show any obvious clinical disease or illness during the study or any gross lesions at necropsy. 

### 3.5. Serum Neutralization

**Figure 5:**
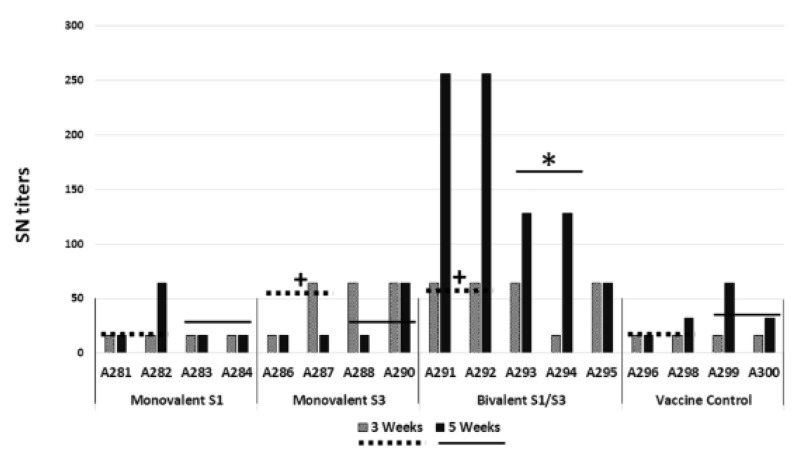
SN antibody titers of individual birds of different groups at 3 and 5 weeks of age in different groups. MonovalentS1, PICV-based TARV vaccine carrying monovalent PICV-SKM121 S1 (codon-optimized) sequence; MonovalentS3, PICV-based TARV vaccine carrying monovalent PICV-SKM121 S3 (codon-optimized) sequence; Bivalent S1/S3, PICV-based TARV vaccine carrying bivalent PICV-SKM121 S1/S3 (codon-optimized) sequences; Vaccine Control, PICV vaccine with no insert (control). A281 to A300 represent tags on individual birds. The dotted lines represent the mean values at 3 weeks old and the solid lines represent the mean values at 5 weeks old. The values of Monovalent S3 and Bivalent S1/S3 groups were significantly higher than the other two groups at 3 weeks old (+), and the values of the Bivalent S1/S3 group were significantly higher than the other three groups at 5 weeks old (*) using the Mann–Whitney U test at *p* < 0.05.

At 3 weeks of age, three of four sera from birds inoculated with the monovalent PICV-based TARV-S3 vaccine and four of five sera from birds inoculated with the bivalent PICV-based TARV S1/S3 vaccine showed serum neutralizing (SN) antibody titers of 64, which were significantly higher (*p* < 0.05) than the other two groups (Figure 5). At 5 weeks of age, only one of three birds inoculated with monovalent PICV-based TARV-S1, monovalent PICV-based TARV-S3, and PICV-control had an SN antibody titer of 64, while all five sera from birds inoculated with the bivalent vaccine had high SN antibody titers ranging from 64 to 256, which were significantly higher (*p* < 0.05) than the other three groups (Figure 5). Individual bird data show that there was a remarkable increase in the SN antibody titers of birds inoculated with the bivalent PICV-based TARV-S1/S3 vaccine at 5 weeks of age than the other groups (Figure 5).

## 4. Discussion

Using PICV as a vector to express TARV antigenic proteins presents an attractive alternative to the use of live attenuated TARV vaccines that may result in the emergence of mutant TARV strains. Recombinant PICV expressing the hemagglutinin and nucleoprotein genes of influenza initiated humoral and cell-mediated immune responses and provided full protection against lethal influenza in mice [16]. Several subunit vaccines reported against chicken reovirus and duck reovirus involve expression of the σC protein alone or in combination with σB proteins [12,13,14,15]. The purpose of this study was to develop recombinant PICV-based TARV vaccines that carry S1 and/or S3 genes of turkey arthritis reoviruses. We successfully recovered recombinant PICV after insertion of TARV genomic segments in the PICV plasmids, detected the inserted genes, and confirmed the expression of recombinant antigenic proteins. 

A total of 12 different recombinant PICVs were developed. We used the wild-type genes from three different TARV isolates in addition to codon-optimized genes from one TARV isolate in an effort to find an optimum TARV candidate to be used in developing a vaccine. The recovered PICV recombinants grew well in BHK-21 cells but showed minimal growth on QT-35 and LMH cells as determined by the expression of GFP in recombinant PICV that contained each of the TARV genes and the GFP gene. No GFP gene was inserted in the bivalent recombinant PICV (containing both S1 and S3 genes of TARV) and, hence, they could not be subjected to the green fluorescence test. We assume that these viruses grew the same way as the monovalent viruses because their plasmids and transfection were done under the same conditions. The detection of the inserted genes and the protein expression was helpful in determining the successful rescue and growth of the bivalent PICV-based TARV vaccines. The use of RT-PCR to detect the inserted TARV genome in recovered viruses helped in determining the success of recovering recombinant PICV-based TARV vaccines that contained TARV S1 and/or S3 genes. 

To confirm the expression of reoviral antigenic proteins by the recombinant PICV-based TARV vaccines, we used the direct fluorescent antibody technique (DFA) using FITC-conjugated anti-avian reovirus antibodies. The FA procedure included dehydration and long fixation with acetone. These two steps helped eliminate GFP fluorescence and increased permeabilization of the cell membrane. The permeabilization of the cell membrane enabled the entrance of the FITC-conjugated antibody to reach the intracellular recombinant viral proteins. Although we did not have a quantitative method to measure the amount of the expressed protein, we could subjectively observe that the fluorescence produced by viruses recombined with SKM121 genes was more than that produced by SKM73 and SKM95. The bivalent PICV-based TARV vaccines that contained codon-optimized S1 and S3 of SKM121 showed the best fluorescence, indicating the growth of these viruses, which did not have any GFP fluorescence after transfection.

Since codon-optimized PICV-based TARV SKM121 vaccines showed the best expression of TARV S1 and/or S3 proteins, we used these vaccines for a pilot in vivo experiment. PICV without an insertion of TARV gene was used as a control. The in vivo experiment was to determine the safety and humoral response generated by the recombinant vaccines in turkey poults. Turkey poults were vaccinated with the same dose of vaccine (0.3 mL, 3 × 10^5^ PFU/mL) via oral and intranasal route using a prime-boost strategy. Poults were primed at 1 week of age because birds are susceptible to avian reovirus infection in the early days of their life [22,23]; hence, vaccination strategies are designed to provide passive immunity from maternal antibodies by vaccinating breeders or by actively immunizing young bids with a live vaccine [24]. Priming at 1 week of age was also considered to avoid vaccination shock and poor intestinal immunity in day old birds [24]. Booster dose was given intranasally at 3 weeks of age, targeting the coarse spray administration with the same vaccine as previously described [25]. The booster vaccination was done after 2 weeks of priming because the recombinant PICV-based vaccine needs 2–3 weeks to provoke the best immune responses. Immunogenicity of the codon-optimized bivalent PICV-based TARV SKM121 vaccine was demonstrated by the production of high SN antibody titers. In future in vivo experiments, we plan to further characterize the effect of the dose of the recombinant PICV-based TARV vaccines on antibody titer and response to reovirus challenge in turkeys, as well as to study the efficacy of protection of the PICV-based TARV vaccines against heterologous and homologous challenge viruses.

## Figures and Tables

**Table 1:** List of primers used for full-length open reading frame (ORF) amplification of S1 and S3 segments of turkey arthritis reovirus. The bold sequence part is specific and complementary for the turkey reovirus genes. The italicized sequence part is a tag sequence. The lowercase sequence part is a restriction enzyme (RE) site. The underlined sequence part is the Kozak sequence. TARV, turkey arthritis reovirus; F, forward; R, reverse; HA, hemagglutinin.

Scheme 5.	Name	Sequence (5’ to 3’)	Number of Bases	Position of Primer on Viral Template (ORFs)	Expected PCR Product Size (Including Primer Sequences)	RE Site	Tag
1	TARV-S1 F	CGATgctagcGCCACC**ATGG****CCGCTCTAACTCCGTC**	36	1–20	1031 bp	*Nhe*I	-
2	TARV-S1 R	ATCGctcgagTTA*CTTGTCGTCATCGTCTTTGTAGTC***GGTGTCGATGCCCGTACGCA**	57	978–959		*Xho*I	FLAG
3	TARV-S3 F	CGATgctagcGCCACC**ATGG****AGGTACGTGTGCCAAACTTTC**	41	1–25	1157 bp	*Nhe*I	-
4	TARV-S3 R	ATCGctcgagTTA*AGCGTAATCTGGAACATCGTATGGGTA***CCAACCACACTCCATAAAAGTCAG**	64	1101–1078		*Xho*I	HA

**Table 2:** Pichinde virus (PICV) recombinant plasmids used to generate PICV-based TARV vaccines. GPC, glycoprotein; MCS, multiple cloning site; NP, nucleoprotein.

Serial Number of Recombinant PICV-Based TARV Vaccines	Strains of Turkey Arthritis Reovirus	Recombinant PICV-Based TARV Vaccine Type	Insert in Plasmid 1 pP18S1-GPC/MCS	Insert in Plasmid 2 pP18S2-MCS/NP
1	SKM73 *	Monovalent	GFP ^a^	S1 ^+^ wild-type
2		Monovalent	S3 ^+^ wild-type	GFP
3		Bivalent	S3 wild-type	S1 wild-type
4	SKM95 *	Monovalent	GFP	S1 wild-type
5		Monovalent	S3 wild-type	GFP
6		Bivalent	S3 wild-type	S1 wild-type
7	SKM121 *	Monovalent	GFP	S1 wild-type
8		Monovalent	S3 wild-type	GFP
9		Bivalent	S3 wild-type	S1 wild-type
10	SKM121 *	Monovalent	GFP	S1 codon-optimized
11		Monovalent	S3 codon-optimized	GFP
12		Bivalent	S3 codon-optimized	S1 codon-optimized
13		Control	GFP	GFP

^a^ GFP = green fluorescence protein. ^+^ S1 and S3 are genes inserted into PICV plasmids. * SKM73, SKM95, and SKM121 are designations of turkey arthritis reoviruses whose S1 and S3 genes were inserted into PICV plasmids.

## Data Availability

The data presented in this study are openly available in FigShare at 10.6084/m9.figshare.13980386.

## Supplementary Materials

The following are available online at https://www.mdpi.com/2076-0817/10/2/197/s1, Figure S1: Codon optimized and wild-type sequences of TARV sigma C and sigma B genes.
