# Supplementary material for: Development of a Recombinant Pichinde Virus-Vectored Vaccine against Turkey Arthritis Reovirus and Its Immunological Response Characterization in Vaccinated Animals
Source: Pathogens. 2021 Feb 13;10(2):197. doi: 10.3390/pathogens10020197 (PMC7918942; doi:10.3390/pathogens10020197)
Supplement: Supplementary file 1 [file pathogens-10-00197-s001.pdf]

Figure S1: Codon optimized and wild-type sequences of TARV sigma C and sigma B genes

Codon optimized sequence of SKM121-S1 segment-sigma C

ATGGCCGCTTTAACACCTAGCCAGAGGAGGGAGGTGGTGGGCCTCATTTTATCTTTAACCTCCAATGCCAACACCTCTTGTGGAGA
TTTAGCCCCCATTTTAGAGAGACTGGTGAGGCTGGACAGCACCGTGAAACTGCTGACCGAATCCGTGGGCGTGCTGAGCCAGAAGG
TGAGCGCTTTCGAGAGCTCTTTAAAGGACACCAACTCCTCTTTAAGCAAAGTCACAGCCAGCATGACAGAACTGTCCGGCGAGCTG
CACCAACTGCACGCCTCTTTAGGCGACGTGGATACCAGCGTGCTGGACATGTCCGCCACTTTAACCAAGCACGGAAATTTAATCAC
CGCTTTACAGACCAGCGTGCAGAACAACGCTAACGCCATCTCCAATTTAAAGAGCAGCGTGTCCGCTTTAGGACTGAGCGTGACAG
ACATCGACGGTCGTCTGAAGGCTGTGGAGAGCGGCAGCGGCAGCCCTTTAAAGTTCTCCACCCCTCTGAAGCTGGACGGCGGCGTG
GTGTCTTTAGACATGGACCCCTACTTCTGCAGCGACAACCACGTGCTGACCAGCTATTCCACCGACGCCCAGCTGATGCAGTTCCA
GTGGCTCGCTAGAGGCGCTGATGGCAGCTCCGGAAGCGTCGACATGCTGGTGAACGCCCACTGTCATGGCAGAAGGACCGACTACA
TGATGTCCACCACCGAGAATTTAACCATCACCGGCAACAGCACCAGCCTCGTGTTCAATTTAGACTACATCACCAAGCCCCCCACC
GACATGTCCAGACTGATTCCTAGAGCTGGCTTCCAAGCCGCCAGCTTCCCCGTGGATGTGAGCTTCACAAGAGACGACACCACACA
CGCCTACCAAGTGTACGGCACCTTCACCAGCCCTCGTGTGTTCAAGATCACCTTTTTAACTGGTGGCGCTGGCACCGCCAATTTAA
GATTTTTAACAGTGAGGACCGGAATCGACACC

Wild-type sequence of SKM121-S1 segment-sigma C

ATGGCCGCTCTAACTCCGTCACAGCGAAGAGAAGTCGTGGGATTGATACTCTCCTTGACTTCGAACGCGAATACAAGTTGTGGAGA
TTTGGCGCCGATACTCGAACGGTTGGTTAGGCTAGACTCCACTGTTAAGTTGTTGACTGAATCTGTGGGTGTCCTGTCTCAGAAGG
TATCTGCCTTCGAGTCTAGTTTAAAAGACACTAATTCTTCCTTAAGTAAGGTTACTGCATCAATGACTGAGCTGTCGGGTGAACTT
CATCAACTCCATGCGTCCCTTGGTGACGTTGACACGTCGGTATTGGACATGTCCGCGACTTTAACTAAGCATGGTAATTTGATAAC
CGCTCTGCAGACTTCCGTGCAGAATAATGCGAATGCGATTAGTAATTTGAAGAGCAGTGTGTCTGCTCTGGGTCTAAGTGTTACTG
ATATTGATGGGCGTTTGAAGGCTGTTGAATCGGGTTCGGGCTCCCCACTGAAGTTCTCAACTCCTCTGAAGCTTGACGGTGGCGTA
GTGTCTTTAGACATGGACCCGTACTTTTGTTCAGATAATCATGTCCTAACCTCATATTCTACAGACGCTCAACTCATGCAATTTCA
GTGGCTGGCGCGTGGTGCAGATGGATCTTCTGGTTCCGTCGATATGCTTGTTAACGCGCACTGTCACGGGCGCCGAACTGATTACA
TGATGTCGACTACAGAGAACCTCACTATTACTGGTAACTCTACCTCCTTAGTTTTCAACCTAGATTACATTACCAAGCCGCCCACT
GACATGTCACGTCTCATTCCACGCGCTGGATTTCAAGCTGCTTCTTTCCCGGTGGATGTGTCATTTACTCGTGATGACACGACGCA
CGCTTACCAGGTATATGGTACATTCACTAGCCCGCGCGTCTTTAAGATCACATTTCTCACTGGGGGGGCTGGGACTGCGAACCTCC
GTTTCTTGACCGTGCGTACGGGCATCGACACC

Codon optimized sequence of SKM121-S3 segment-sigma B

ATGGAGGTGAGGGTGCCCAACTTCCACTCCTTCGTCGAGGGCATCACCTCCTCCTATATCAGAGCCCCCGCTTGTTGGAACGCCAA
GACCATGTGGGATGTGGAGACCTTTCATTTACCCGACGTGATCAAGGTGGGCAACGCCTACTGTTGCTCCCAGTGCTGCGGCGTGC
TGTACTACGGAGCTCCCCCCAGCGATGGCAACTACTTCCCCCACCACAAGTGCCACCAGCAGCAGTATCGTTCCGACACCCCTTTA
CTGAGATACGTGAGAATCGGAAGGACCACCGAGCATTTACTGGATCAGTACGCCGTGACTTTACAGACCATTGCCGACTATTACGA
CGAGACCTCCCATCGTGTGGCTGATGACAGCGAGGAGGACACCATCGCCGCTTTAGATATCGTGACTCGTACCGAGAGCATCAGAA
GCGACCAAGCTGTGGACGCCGACTTCTGGACCTACCCTTTAGAGAGGAGGAGCGATGACTCTCGTAGGGATATCGCCGCCAGCATC
TGGACCATGATCGACGCCAGCGCTCGTAGCTTCACTTTACCCGACTGTCTGGTGAGCCCTAGCCTCCACTCTCGTCACGTGTTCGA
CCAGATGCTGACCACAACCAGCATCTACGATGTGGCCGCTTCCGGCAAGACAGCTCGTTTCTCCCCCATGGTGGCCGCTCTGCCTC
AAAGAGCCGCTGGCCCCATCACTTTACCCGATGCCGACCCTCTGGACGGCGTGGCTACCTTCTGGAGCCCCCAGTTCGCCCTCTCC
CCTATGATCGGCGGCGTGGGCATTACCGGACAGTACGCTCGTGAGTCCTACCACCACGTGGGCCACCCCGTGATCGGAAGCGGCAA
GAAGGTCTCCCACTATCGTAATTTATTCATGGATGCTTGGAGAGGATGGAGCAAGTCCTCCTTCACATGTGCCGCCGGTTTAGAAC
CCGCTGAATGCGAGAGCAGACTGAGGGGCCATGCTCGTACCATGCTGGGCAGATCTTTACCCGGCGTGTGTGACTGCGGCAGCGAA
GCTCAGCCTAGGACCGCCCCTAGCTCTTTACAGAAGGCCACCAAGCTGACCTTCATGGAGTGTGGCTGG

Wild-type sequence of SKM121-S3 segment-sigma B

ATGGAGGTACGTGTGCCAAACTTTCACTCTTTTGTTGAAGGCATTACATCGAGTTACATTAGAGCTCCTGCTTGCTGGAATGCGAA
AACGATGTGGGATGTTGAGACTTTCCATCTTCCGGATGTAATCAAGGTCGGTAATGCTTATTGTTGCTCGCAATGCTGTGGAGTGT
TGTATTATGGAGCGCCTCCCTCTGATGGAAACTATTTTCCTCACCACAAATGTCATCAACAGCAGTATCGCAGTGACACCCCTCTC
TTGCGTTACGTCAGAATTGGGCGCACAACTGAGCATCTTCTGGACCAATACGCCGTCACCCTCCAGACTATCGCTGATTATTATGA
TGAGACGAGCCATCGCGTCGCCGATGATTCTGAAGAAGACACCATAGCTGCCCTTGACATTGTGACGAGAACTGAATCTATCCGTA
GTGACCAAGCCGTGGATGCTGATTTTTGGACGTACCCCCTCGAACGACGATCTGACGACTCCCGGCGTGACATCGCTGCTTCAATT
TGGACGATGATTGATGCTTCGGCGCGTAGTTTTACGTTGCCTGATTGTCTTGTCTCCCCGTCGCTCCACTCACGTCATGTTTTTGA
TCAAATGCTGACTACTACGTCTATTTATGACGTCGCTGCCTCAGGAAAGACGGCTAGATTTAGTCCAATGGTGGCTGCTCTTCCAC
AACGCGCTGCCGGTCCGATTACACTTCCAGACGCTGACCCGCTTGATGGAGTAGCCACGTTCTGGTCACCTCAGTTTGCTCTTTCA
CCTATGATTGGTGGCGTTGGCATTACAGGGCAATACGCGCGTGAATCATATCACCATGTGGGACACCCTGTCATTGGGAGCGGAAA
GAAAGTATCGCACTACCGCAACCTCTTCATGGACGCGTGGCGTGGGTGGTCGAAGTCGTCGTTTACGTGTGCTGCTGGTCTGGAAC
CTGCTGAGTGTGAGTCTCGTCTTCGTGGGCACGCGAGAACGATGCTCGGTCGCTCGTTGCCTGGAGTATGCGACTGTGGATCCGAG
GCTCAGCCCAGGACCGCGCCGTCTTCTCTGCAAAAGGCTACTAAACTGACTTTTATGGAGTGTGGTTGG
